# Defining clinically meaningful pain reduction after spinal injections using an 11-point numerical rating scale and patient-reported relative reduction of pain

**DOI:** 10.1371/journal.pone.0361475

**Published:** 2026-10-26

**Authors:** Yoonah Do, Wookon Son, Youngjune Kim, Choong Guen Chee, Yusuhn Kang, Eugene Lee, Yeo Ju Kim, Joon Woo Lee

**Affiliations:** 1 Department of Radiology, Seoul National University Bundang Hospital, Seongnam, South Korea; 2 Seoul National University College of Medicine, Seoul, South Korea; University of California San Diego, UNITED STATES OF AMERICA

## Abstract

**Background:**

We aimed to establish thresholds for assessing clinically meaningful pain improvement after spinal injections using numerical rating scale (NRS) and patient-reported relative reduction (PRR) of pain.

**Methods:**

This single-center retrospective study included 1,828 patients who received spinal injections at our spinal intervention center and completed a 2-week follow-up telephone interview in 2017. NRS absolute and relative reductions were calculated using the NRS scores before injection and at follow-up. The PRR and 5-point global perceived effect (GPE; 1, completely recovered; 2, much improved; 3, slightly improved; 4, no change; and 5, worsened) were recorded. The mean ± standard deviation (SD) of NRS absolute reduction, NRS relative reduction, and PRR in the GPE 2 and 3 subgroups were calculated. Receiver-operating-characteristic curve analysis was used to determine the cutoffs for NRS absolute and relative reduction and PRR in predicting the substantial clinically important difference (SCID, GPE 1–2) and minimal clinically important difference (MCID, GPE 1–3).

**Results:**

The mean ± SD of NRS absolute and relative reduction, and PRR in the GPE 2 and GPE 3 were 4.5 ± 1.8, 65.4% ± 21.1%, and 67.7% ± 14.8% and 2.4 ± 1.4, 36.8% ± 22.5%, and 35.7% ± 16.1%, respectively. The cutoffs for predicting SCID and MCID were NRS absolute reduction of ≥3 and ≥1, NRS relative reduction of ≥50% and ≥10%, and PRR of ≥50% and ≥20%, respectively.

**Conclusions:**

The NRS absolute and relative reduction and PRR thresholds were established to differentiate SCID and MCID in terms of pain improvement after spinal injections.

## Introduction

Spinal injection is widely utilized to manage symptoms of spinal origin, including radicular pain. The number of procedures performed annually has been steadily increasing [1]. The severity of pain of spinal origin is commonly assessed using the numerical rating scale (NRS) or visual analog scale. These tools are essential for evaluating the patient’s condition and treatment responses.

Clinically, a satisfactory reduction in pain is typically defined as a decrease of ≥50% in pain scores. This criterion was initially proposed in a 1991 study by Johns Hopkins University [2,3]. However, applying a 50% threshold across all patient populations has faced substantial criticism [4,5]. Several studies have reported varying thresholds for clinically significant pain reductions. For example, Farrar et al. found that in patients with cancer pain treated with fentanyl, a clinically significant reduction in pain was associated with a ≥ 33% decrease in pain scores [6]. In an analysis of 10 studies examining pregabalin for various pain etiologies, Farrar et al. reported that a 30% reduction in pain scores was clinically meaningful [7]. Similarly, Cepeda et al. observed that in patients with acute pain, a reduction of approximately 35% in pain scores was linked to significant improvement [8].

Research on spinal injections often defines a significant reduction in pain as a decrease of ≥50% [9–14]. This threshold, initially established by Manchikanti et al. in a series of randomized controlled trials in 2012, has been widely adopted in subsequent studies without much scrutiny. However, evidence supporting the 50% reduction as a reliable indicator of clinically significant pain improvement remains scarce. Furthermore, although most spinal disorders frequently involve both localized pain (e.g., low back or posterior neck pain) and radiating pain, limited attention has been given to evaluating radiating pain specifically in this context.

Recent studies have increasingly emphasized a patient-centered approach in interpreting subjective outcomes, highlighting the importance of measuring satisfaction from the patient’s perspective [15,16]. The concept of minimal clinically important difference (MCID) is gaining recognition, as it reflects the smallest change in patient-reported scores that is deemed meaningful following a clinical intervention. Moreover, a growing need exists to measure not only minimal changes but also the extent of clinically significant effects, defined as substantial clinically important differences (SCID). However, no previous studies have established the exact cutoff values for MCID and SCID following spinal injections.

Therefore, this study aimed to assess the clinically meaningful pain improvement two weeks after spinal injection based on the NRS scores and patient-reported relative reduction (PRR) of pain.

## Materials and methods

This retrospective study was conducted at an urban university hospital in South Korea. The requirement for written informed consent was waived by our Institutional Review Board owing to the retrospective nature of this study (B-2411-934-103). This study used de-identified data accessed for research purposes from 1/11/2024–31/12/2024, and the authors did not have access to any information that could identify individual participants during or after data collection.

### Patient inclusion

In 2017, 2,824 patients were initially referred to our spinal intervention center and received spinal injections. We excluded patients who did not participate in a telephone interview conducted two weeks post-injection, those who did not assess the global perceived effect (GPE) of the spinal injection, those who did not respond to the PRR of pain scale, and those who provided invalid responses on the NRS two weeks post-injection.

### Questionnaire

All patients visiting our spinal intervention center were required to complete a structured questionnaire to systematically evaluate their symptoms (S1 Appendix). The questionnaire assessed the duration of pain onset (i.e., acute [< 1 month], subacute [1–6 months], or chronic [> 6 months]), the degree of pain using an 11-point NRS (0 indicating no pain and 10 indicating the worst pain imaginable), and pain location (categorized as lower back pain, pain radiating to the legs, posterior neck pain, pain radiating to the arms, and upper back pain).

### Spinal injection

Magnetic resonance imaging is commonly used in conjunction with patient signs and symptoms to determine the source of pain. Spine interventional radiologists evaluated the contraindications for spinal injections, including infection and coagulopathy. Specifically, a prothrombin time international normalized ratio of >1.5 or a platelet count of ≤50,000/μL was considered a contraindication for interlaminar and transforaminal injections [17]. Although no specific contraindications were set for facet joint injections in patients with coagulopathy, a smaller 25-gauge needle was used to minimize the risk of hemorrhage. Interlaminar injections are indicated for central zone pathologies, such as spinal stenosis or herniated intervertebral disc (HIVD), whereas transforaminal injections are indicated for subarticular, foraminal, or extraforaminal pathologies. Caudal injections are considered for patients who are not candidates for interlaminar or transforaminal injections, such as those with multilevel pathologies or who lack epidural fat. Intra-articular facet joint injections were considered in patients with facet osteoarthritis, although supporting evidence for this practice remains limited [18]. Additionally, facet joint injections were employed for osteoporotic compression fractures, as recent studies indicated that pain may arise from the facet joints [19,20]. In patients with osteoporotic compression fractures, spinal injections were administered when severe pain persisted despite appropriate conservative treatment and when surgical intervention was not indicated. For post-lumbar surgery syndrome, the choice of injection technique was determined based on the specific pathology of each patient, including spinal stenosis, HIVD, instability, olisthesis, or facet joint issues. Bilateral facet joint injections are indicated in patients with instability, olisthesis, or facet osteoarthritis [21]. Caudal injections have also been used to manage post-lumbar surgery syndromes.

The equipment and drug dosages for spinal injections were consistent with those published elsewhere [21,22]. For cervical interlaminar and transforaminal injections, we used 10 mg (2 mL) of 5 mg/mL dexamethasone disodium phosphate. For cervical facet joint injections, we used 20 mg (0.5 mL) of 40 mg/mL triamcinolone acetonide. To mitigate the potential complications associated with inadvertent intradural injections of local anesthetics, ropivacaine or other local anesthetics were not used for cervical injections. For lumbar interlaminar and transforaminal injections, we used 10 mg (2 mL) of 5 mg/mL dexamethasone disodium phosphate plus ropivacaine hydrochloride (7.5 mg [1 mL] for interlaminar injections and 3.75 mg [0.5 mL] for transforaminal injections). For lumbar facet joint injections, we used 20 mg of triamcinolone acetonide mixed with 3.75 mg (0.5 mL) of ropivacaine hydrochloride per facet joint, with a maximum of two facet joints injected simultaneously. For caudal injections, we used 10 mg (2 mL) of 5 mg/mL dexamethasone disodium phosphate combined with 3.75 mg (0.5 mL) of ropivacaine hydrochloride.

### Telephone interview

Routine telephone interviews were conducted 2 weeks post-injection to assess the effects of the spinal injection, focusing on GPE, current pain level using an 11-point NRS, and PRR. The GPE was classified using a 5-point scale: 1, completely recovered; 2, much improved; 3, slightly improved; 4, no change; and 5, worsened. The NRS absolute reduction was defined as the absolute reduction at the pain site showing the largest absolute reduction among the evaluated sites. The NRS relative reduction was defined as the relative reduction at the pain site showing the largest relative reduction among the evaluated sites. PRR was defined as the degree of pain reduction after the injection, expressed as a percentage based on the pain level before the injection.

### Statistical analysis

We compared baseline characteristics between included and excluded patients. Continuous variables were compared using Welch’s t-test, and categorical variables were compared using the chi-square test. In addition, pain-response outcomes of the included patients were also summarized. The mean ± standard deviation (SD) values of NRS absolute reduction, NRS relative reduction, and PRR in the GPE 1 (completely recovered) to 3 (slightly improved) subgroups were calculated.

Receiver operating characteristic (ROC) curve analyses were performed to identify the optimal cutoff values for NRS absolute reduction, NRS relative reduction, and PRR indicating clinically significant pain reduction. The areas under the receiver operating characteristic curve (AUC) were calculated and compared using DeLong’s nonparametric method [23]. The optimal cutoff value was defined as the point on the ROC curve closest to the top left corner, determined by incrementing the NRS absolute reduction by 1 and NRS relative reduction and PRR by 10%. The analyses were repeated using three different definitions of pain reduction: i) complete recovery, defined as a GPE score of 1 (completely recovered); ii) SCID, defined as GPE scores of 1 (completely recovered) and 2 (much improved); and iii) MCID, defined as GPE scores of 1 (completely recovered) to 3 (slightly improved). The sensitivity and specificity of each cutoff value were then calculated. Subgroup analysis was performed to identify potential associations between the subgroups and the diagnostic performance of each cutoff value for predicting SCID (i.e., GPE scores of 1 [completely recovered] and 2 [much improved]). The variables tested included age groups (≤34, 35–49, 50–64, and ≥65 years), sex, duration of pain onset (<1 month, 1–6 months, > 6 months, and unknown), pain site with maximal NRS reduction which is the pain site showing the greatest absolute NRS reduction from baseline to follow-up (low back pain, pain radiating to the legs, posterior neck pain, pain radiating to the arms, and upper back pain), baseline NRS at the maximal-reduction site (severe, NRS 7–10; moderate, NRS 4–6; and mild, NRS 0–3), and types of spinal injection (lumbar interlaminar, lumbar transforaminal, caudal, lumbar facet, cervical interlaminar, cervical transforaminal, cervical facet, and others). All statistical analyses were performed using R version 4.3.3 (the R Foundation for Statistical Computing, Vienna, Austria). A two-sided *P* value of <0.05 was considered significant.

## Results

Among the 2,824 patients referred to our spinal intervention center and received spinal injections in 2017, 996 were excluded after a 2-week telephone interview. Specifically, 960 patients did not participate in the interview, 14 provided inappropriate GPE scores, 3 provided incorrect responses to the PRR assessment, and 19 failed to accurately report their current pain level using the 11-point NRS (Fig 1). Thus, only 1,828 patients were included in the final analysis. Baseline characteristics between included and excluded patients are presented in Table 1. The proportion of female patients was higher among excluded patients than among included patients (57.1% vs. 52.8%, *P* = 0.030). No significant differences were observed in age, onset of pain, dominant pain site, initial numerical pain NRS score, or type of spinal injection.

**Table 1:** Baseline characteristics of included and excluded patients.

	Included (n = 1,828)	Excluded (n = 996)	*P* value^1^
Age (years)	61 ± 14	61 ± 15	0.696
≤34	73 (4.0)	64 (6.4)	
35–49	283 (15.5)	148 (14.9)	
50–64	653 (35.7)	327 (32.8)	
≥65	819 (44.8)	457 (45.9)	
Sex			0.030^*^
Female	965 (52.8)	569 (57.1)	
Male	863 (47.2)	427 (42.9)	
Onset of pain			0.239
<1 month	227 (12.4)	113 (11.3)	
1–6 months	708 (38.7)	425 (42.7)	
>6 months	879 (48.1)	451 (45.3)	
Unknown	14 (0.8)	7 (0.7)	
Dominant pain site^2^			0.374
Lower back	630 (34.5)	326 (32.7)	
Pain radiating to the legs	734 (40.2)	403 (40.5)	
Posterior neck	235 (12.9)	147 (14.8)	
Pain radiating to the arms	225 (12.3)	115 (11.5)	
Upper back	4 (0.2)	5 (0.5)	
Initial numerical pain scale^3^	7.6 ± 1.8	7.6 ± 2.0	0.841
Severe (7–10)	1,363 (74.6)	739 (74.2)	
Moderate (4–6)	422 (23.1)	230 (23.1)	
Mild (0–3)	43 (2.4)	27 (2.7)	
Type of spinal injection			0.922
Lumbar interlaminar	314 (17.2)	162 (16.3)	
Lumbar transforaminal	519 (28.4)	300 (30.1)	
Lumbar facet	288 (15.8)	149 (15.0)	
Caudal	205 (11.2)	100 (10.0)	
Cervical interlaminar	405 (22.2)	230 (23.1)	
Cervical transforaminal	43 (2.4)	25 (2.5)	
Cervical facet	5 (0.3)	2 (0.2)	
Others	49 (2.7)	28 (2.8)	

Data are expressed as numbers of patients and percentages in parentheses for categorical variables and means ± standard deviations for continuous variables.

^1^*P* values for comparing the included patients (n = 1,828) with excluded patients (n = 996) were calculated using Welch’s t-test for continuous variables and the chi-square test for categorical variables.

^2^Dominant pain site was defined as the site with the highest pre-injection NRS score.

^3^Initial numerical pain scale was defined as the highest pre-injection NRS score among all evaluated pain sites.

* P value of <0.05

**Fig 1:**
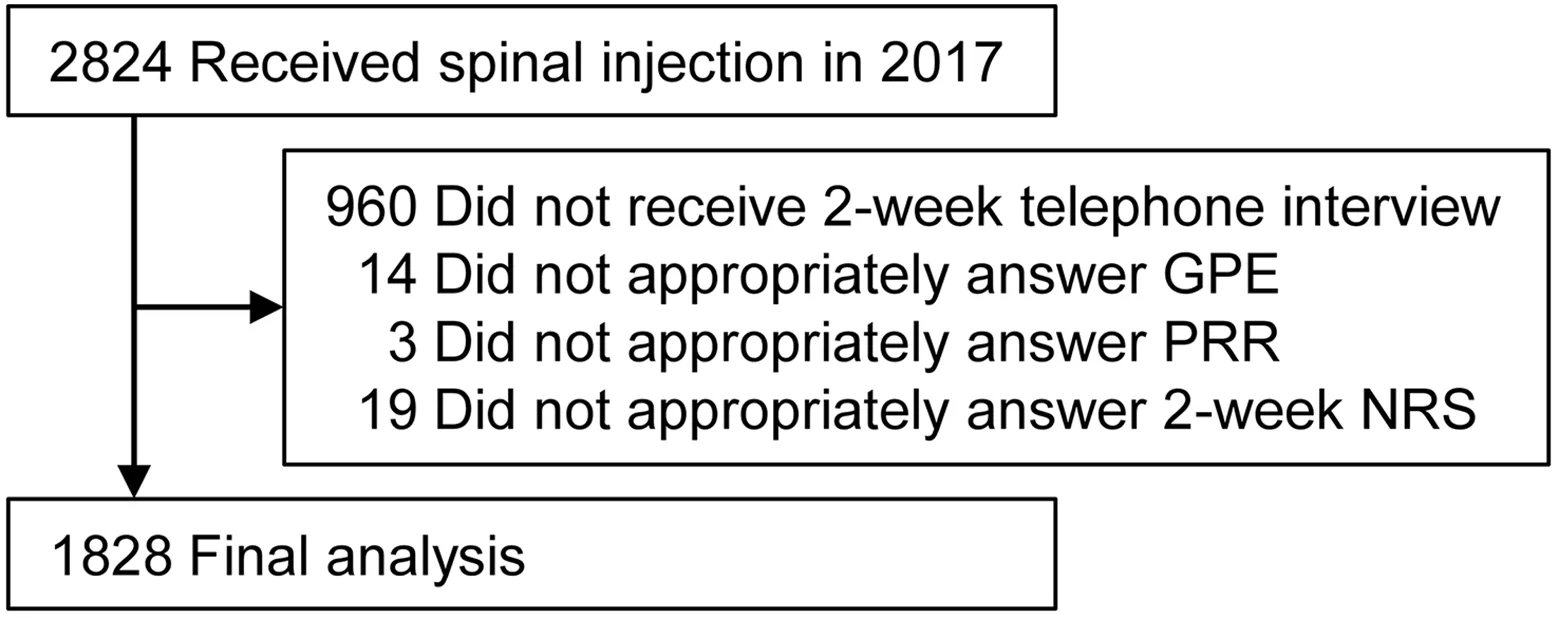
Diagram of the patient inclusion process. Abbreviations: GPE, global perceived effect; PRR, patient-reported relative reduction; NRS, numerical rating scale.

Pain-response outcomes of the included patients are presented in Table 2. The mean ± SD values of NRS absolute reduction, NRS relative reduction, and PRR in the GPE 3 subgroup (slightly improved) were 2.4 ± 1.4, 36.8% ± 22.5%, and 35.7% ± 16.1%, respectively. In the GPE 2 subgroup (much improved), the corresponding values were 4.5 ± 1.8, 65.4% ± 21.1%, and 67.7% ± 14.8%, respectively. In the GPE 1 (completely recovered) subgroup, the mean ± SD values of NRS absolute reduction, NRS relative reduction, and PRR were 6.1 ± 2.3, 100.0% ± 0.0%, and 99.6% ± 1.9%, respectively. Box-whisker plots of the NRS absolute reduction, NRS relative reduction, and PRR by GPE are shown in Fig 2.

**Table 2:** Pain-response outcomes of the included patients.

Outcomes		Data
Pain site with maximal NRS reduction		
Lower back		882 (48.2)
Pain radiating to the legs		482 (26.4)
Posterior neck		311 (17.0)
Pain radiating to the arms		149 (8.2)
Upper back		4 (0.2)
NRS absolute reduction		2.2 ± 2.3
NRS relative reduction		33.0% ± 34.4%
Patient-reported relative reduction		33.4% ± 31.0%
Global perceived effect		
1, completely recovered		35 (1.9)
2, much improved		486 (26.6)
3, slightly improved		695 (38.0)
4, no change		534 (29.2)
5, worsened		78 (4.3)

Data are expressed as numbers of patients and percentages in parentheses for categorical variables and means ± standard deviations for continuous variables.

**Fig 2:**
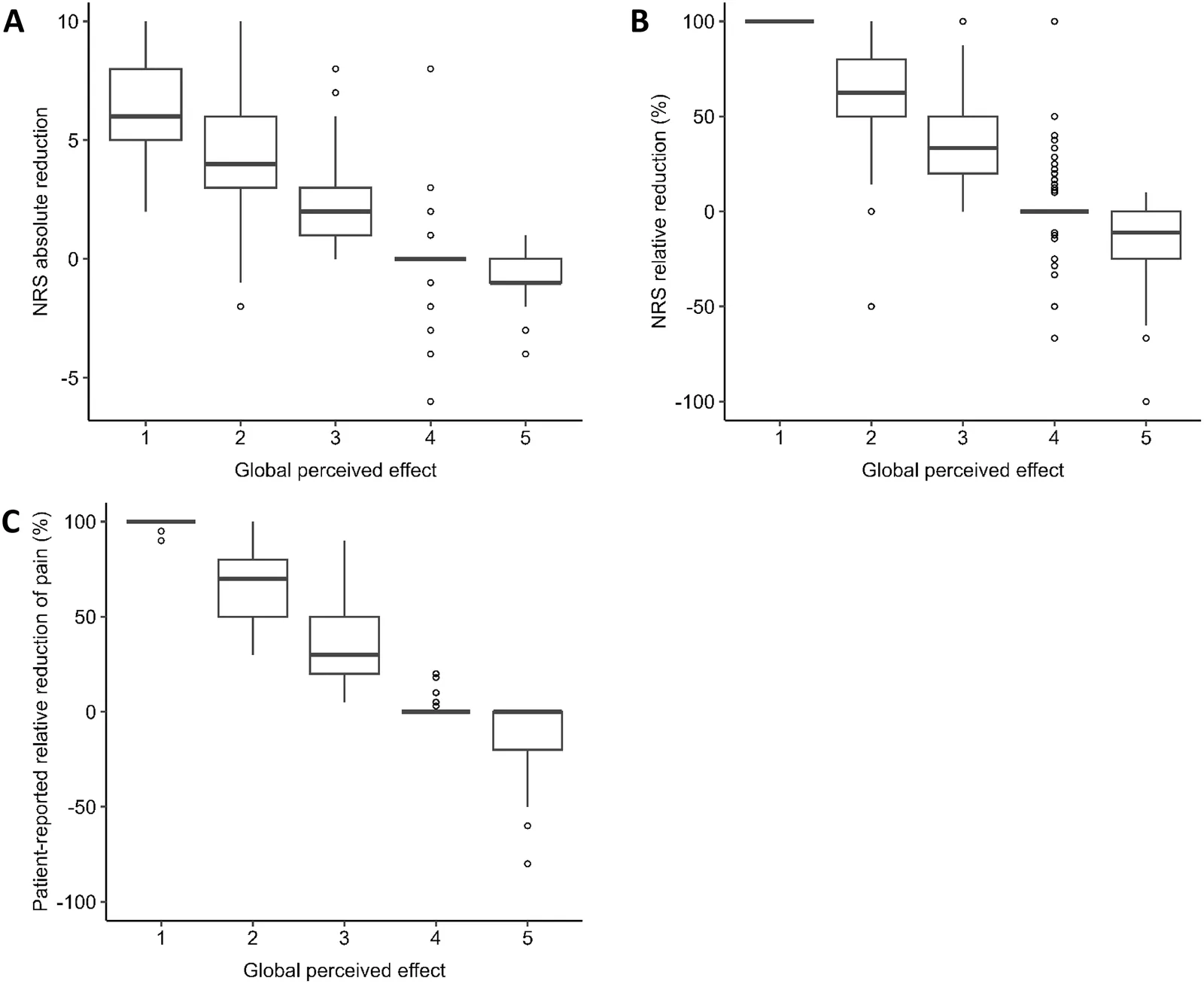
Box-whisker plots of A NRS absolute reduction, B NRS relative reduction, and C patient-reported relative reduction of pain according to global perceived effect. Abbreviation: NRS, numerical rating scale.

ROC curve analyses to identify the optimal cutoff values for NRS absolute reduction, NRS relative reduction, and PRR indicating clinically significant pain reduction are listed in Table 3. NRS relative reduction exhibited a higher AUC for predicting complete recovery (GPE 1 vs. 2–5) (*P* < 0.001) and SCID (GPE 1–2 vs. 3–5) (*P* = 0.043) compared with NRS absolute reduction. However, no significant difference was observed in predicting MCID (GPE 1–3 vs. 4–5) (*P* = 0.361). The PRR demonstrated higher AUC values for predicting complete recovery, SCID, and MCID compared with both NRS absolute and NRS relative reductions (*P* < 0.001). SCID (GPE 1–2) was associated with an NRS absolute reduction of ≥3, an NRS relative reduction rate of ≥50%, and a PRR rate of ≥50%. MCID (GPE 1–3) was associated with an NRS absolute reduction of ≥1, an NRS relative reduction rate of ≥10%, and a PRR rate of ≥20%. Meanwhile, complete pain resolution (GPE 1) was associated with an NRS absolute reduction of ≥5, an NRS relative reduction rate of ≥90%, and a PRR rate of ≥90%.

**Table 3:** Receiver operating characteristic curve analyses to identify the optimal cutoff values for numerical rating scale absolute reduction, NRS relative reduction, and patient-reported relative reduction of pain.

	NRS absolute reduction	NRS relative reduction		Patient-reported relative reduction of pain		
	Data	Data	*P* value^1^	Data	*P* value^1^	*P* value^2^
Substantial clinically important difference						
AUC	0.906 (0.892–0.920)	0.918 (0.904–0.931)	0.043^*^	0.959 (0.952–0.967)	< 0.001^*^	< 0.001^*^
Cutoff	≥3	≥50%		≥50%		
Sensitivity	88.1% (85.0%–90.8%) [459/521]	86.9% (83.7%–89.7%) [453/521]		97.5% (95.8%–98.7%) [508/521]		
Specificity	79.0% (76.7%–81.2%) [1,033/1,307]	84.5% (82.5%–86.5%) [1,105/1,307]		83.2% (81.1%–85.2%) [1,088/1,307]		
Minimal clinicallyimportant difference						
AUC	0.976 (0.969–0.983)	0.974 (0.965–0.982)	0.361	0.998 (0.997–0.999)	< 0.001^*^	< 0.001^*^
Cutoff	≥ 1	≥ 10%		≥ 20%		
Sensitivity	97.5% (96.5%–98.3%) [1,186/1,216]	97.5% (96.5%–98.3%) [1,186/1,216]		96.4% (95.2%–97.4%) [1,172/1,216]		
Specificity	92.5% (90.1%–94.4%) [566/612]	92.5% (90.1%–94.4%) [566/612]		98.7% (97.4%–99.4%) [604/612]		
Completely recovered						
AUC	0.884 (0.840–0.929)	0.969 (0.963–0.974)	<0.001^*^	0.998 (0.996–1.000)	< 0.001^*^	< 0.001^*^
Cutoff	≥5	≥90%		≥90%		
Sensitivity	80.0% (63.1%–91.6%) [28/35]	100.0% (90.0%–100.0%) [35/35]		100.0% (90.0%–100.0%) [35/35]		
Specificity	83.7% (81.9%–85.4%) [1,501/1,793]	93.5% (92.3%–94.6%) [1,677/1,793]		96.3% (95.3%–97.1%) [1,726/1,793]		

Data are expressed as point estimates with 95% confidence intervals in parentheses and numerators/denominators in brackets. The 5-point global perceived effect was divided into three categories: substantial clinically important difference (completely recovered and much improved), minimal clinically important difference (completely recovered, much improved, and slightly improved), and completely recovered. NRS, numerical rating scale; AUC, area under the receiver operating characteristic curve

^1^*P* values for comparing the AUC values with those of NRS absolute reduction were calculated using DeLong’s method.

^2^*P* values for comparing the AUC values with those of NRS relative reduction were calculated using DeLong’s method.

* *P* value of <0.05

The density plots, categorized by GPE scores based on the NRS absolute reduction, NRS relative reduction, and PRR, are shown in Fig 3. The NRS absolute and relative reductions exhibited substantial overlap between the GPE groups. Conversely, PRR demonstrated minimal overlap, suggesting its potential for better diagnostic performance in predicting clinically meaningful pain improvement.

**Fig 3:**
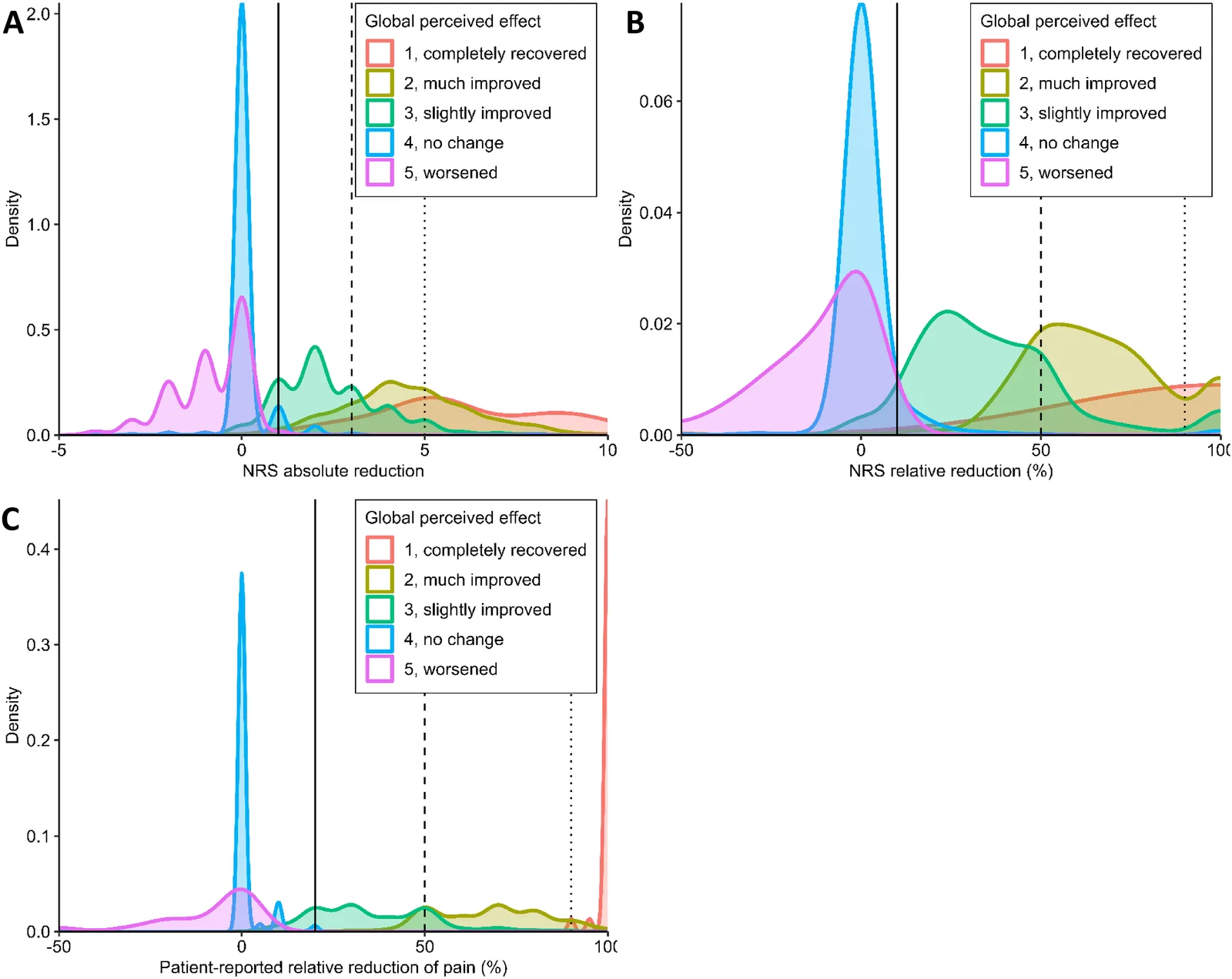
Density plots categorized by global perceived effect based on A NRS absolute reduction, B NRS relative reduction, and C patient-reported relative reduction of pain. Solid lines indicate the cutoff values for improvement (minimal clinical important difference), dashed lines indicate the cutoff values for significant improvement (substantial clinical important difference), and dotted lines indicate the cutoff values for no residual symptoms. Abbreviations: NRS, numerical rating scale.

The results of the subgroup analysis, aimed at identifying the potential associations between the subgroups and the diagnostic performance of each cutoff in predicting SCID (i.e., GPE 1–2), are presented in Table 4. Overall, the sensitivity and specificity remained consistent across various subgroups. However, the sensitivity of NRS absolute reduction was reduced in patients with a mild initial NRS (22.2%), whereas the sensitivity values of NRS relative reduction and PRR were maintained.

**Table 4:** Subgroup analysis of diagnostic performance to determine the cutoff values for predicting the substantial clinically important difference after spinal injection.

	NRS absolute reduction		NRS relative reduction		Patient-reported relative reduction	
	Sensitivity	Specificity	Sensitivity	Specificity	Sensitivity	Specificity
Age (years)						
≤34	95.0% (75.1%–99.9%) [19/20]	75.5% (61.7%–86.2%) [40/53]	85.0% (62.1%–96.8%) [17/20]	75.5% (61.7%–86.2%) [40/53]	100.0% (83.2%–100.0%) [20/20]	75.5% (61.7%–86.2%) [40/53]
35–49	88.6% (80.1%–94.4%) [78/88]	82.1% (75.9%–87.2%) [160/195]	89.8% (81.5%–95.2%) [79/88]	84.1% (78.2%–88.9%) [164/195]	95.5% (88.8%–98.7%) [84/88]	86.2% (80.5%–90.7%) [168/195]
50–64	88.3% (83.1%–92.4%) [181/205]	77.0% (72.8%–80.8%) [345/448]	86.8% (81.4%–91.1%) [178/205]	80.8% (76.8%–84.3%) [362/448]	97.6% (94.4%–99.2%) [200/205]	82.6% (78.8%–86.0%) [370/448]
≥65	87.0% (81.7%–91.3%) [181/208]	79.9% (76.5%–83.0%) [488/611]	86.1% (80.6%–90.5%) [179/208]	88.2% (85.4%–90.7%) [539/611]	98.1% (95.1%–99.5%) [204/208]	83.5% (80.3%–86.3%) [510/611]
Sex						
Female	92.2% (88.1%–95.1%) [235/255]	77.3% (74.1%–80.4%) [549/710]	88.6% (84.1%–92.2%) [226/255]	86.3% (83.6%–88.8%) [613/710]	98.0% (95.5%–99.4%) [250/255]	83.0% (80.0%–85.7%) [589/710]
Male	84.2% (79.3%–88.4%) [224/266]	81.1% (77.7%–84.1%) [484/597]	85.3% (80.5%–89.4%) [227/266]	82.4% (79.1%–85.4%) [492/597]	97.0% (94.2%–98.7%) [258/266]	83.6% (80.4%–86.5%) [499/597]
Onset of pain (month)						
<1	91.2% (83.4%–96.1%) [83/91]	74.3% (66.1%–81.4%) [101/136]	89.0% (80.7%–94.6%) [81/91]	81.6% (74.1%–87.7%) [111/136]	96.7% (90.7%–99.3%) [88/91]	78.7% (70.8%–85.2%) [107/136]
1–6	87.2% (81.9%–91.4%) [184/211]	77.5% (73.5%–81.1%) [385/497]	85.3% (79.8%–89.8%) [180/211]	82.5% (78.9%–85.7%) [410/497]	98.6% (95.9%–99.7%) [208/211]	80.9% (77.1%–84.3%) [402/497]
>6	87.5% (82.3%–91.6%) [189/216]	81.4% (78.3%–84.3%) [540/663]	87.5% (82.3%–91.6%) [189/216]	86.9% (84.1%–89.4%) [576/663]	96.8% (93.4%–98.7%) [209/216]	86.3% (83.4%–88.8%) [572/663]
Unknown	100.0% (29.2%–100.0%) [3/3]	63.6% (30.8%–89.1%) [7/11]	100.0% (29.2%–100.0%) [3/3]	72.7% (39.0%–94.0%) [8/11]	100.0% (29.2%–100.0%) [3/3]	63.6% (30.8%–89.1%) [7/11]
Pain site with maximal NRS reduction						
Low back pain	86.0% (80.5%–90.4%) [178/207]	83.7% (80.7%–86.4%) [565/675]	87.9% (82.7%–92.0%) [182/207]	87.1% (84.3%–89.5%) [588/675]	97.6% (94.5%–99.2%) [202/207]	86.2% (83.4%–88.7%) [582/675]
Radiating pain to leg	94.1% (89.4%–97.1%) [159/169]	67.7% (62.2%–72.9%) [212/313]	88.8% (83.0%–93.1%) [150/169]	80.8% (76.0%–85.0%) [253/313]	97.0% (93.2%–99.0%) [164/169]	75.1% (69.9%–80.0%) [235/313]
Posterior neck pain	84.4% (75.3%–91.2%) [76/90]	85.5% (80.2%–89.9%) [189/221]	82.2% (72.7%–89.5%) [74/90]	84.6% (79.2%–89.1%) [187/221]	98.9% (94.0%–100.0%) [89/90]	89.1% (84.3%–92.9%) [197/221]
Radiating pain to arm	83.0% (70.2%–91.9%) [44/53]	67.7% (57.4%–76.9%) [65/96]	84.9% (72.4%–93.3%) [45/53]	78.1% (68.5%–85.9%) [75/96]	96.2% (87.0%–99.5%) [51/53]	75.0% (65.1%–83.3%) [72/96]
Upper back pain	100.0% (15.8%–100.0%) [2/2]	100.0% (15.8%–100.0%) [2/2]	100.0% (15.8%–100.0%) [2/2]	100.0% (15.8%–100.0%) [2/2]	100.0% (15.8%–100.0%) [2/2]	100.0% (15.8%–100.0%) [2/2]
Baseline NRS at the maximal-reduction site						
Severe (7–10)	98.6% (96.7%–99.5%) [341/346]	72.5% (69.3%–75.5%) [600/828]	89.0% (85.2%–92.1%) [308/346]	86.4% (83.8%–88.6%) [715/828]	97.7% (95.5%–99.0%) [338/346]	83.1% (80.4%–85.6%) [688/828]
Moderate (4–6)	75.7% (67.9%–82.3%) [112/148]	89.1% (85.5%–92.1%) [336/377]	82.4% (75.3%–88.2%) [122/148]	84.1% (80.0%–87.6%) [317/377]	96.6% (92.3%–98.9%) [143/148]	80.1% (75.7%–84.0%) [302/377]
Mild (0–3)	22.2% (8.6%–42.3%) [6/27]	95.1% (88.9%–98.4%) [97/102]	85.2% (66.3%–95.8%) [23/27]	71.6% (61.8%–80.1%) [73/102]	100.0% (87.2%–100.0%) [27/27]	96.1% (90.3%–98.9%) [98/102]
Type of spinal injection						
Lumbar interlaminar	85.0% (75.3%–92.0%) [68/80]	80.8% (75.1%–85.6%) [189/234]	87.5% (78.2%–93.8%) [70/80]	81.6% (76.1%–86.4%) [191/234]	97.5% (91.3%–99.7%) [78/80]	83.8% (78.4%–88.2%) [196/234]
Lumbar transforaminal	88.8% (82.2%–93.6%) [119/134]	78.7% (74.3%–82.7%) [303/385]	85.1% (77.9%–90.6%) [114/134]	86.8% (83.0%–90.0%) [334/385]	97.8% (93.6%–99.5%) [131/134]	85.5% (81.5%–88.8%) [329/385]
Lumbar facet	92.7% (86.2%–96.8%) [102/110]	71.3% (64.1%–77.9%) [127/178]	92.7% (86.2%–96.8%) [102/110]	80.9% (74.3%–86.4%) [144/178]	98.2% (93.6%–99.8%) [108/110]	71.9% (64.7%–78.4%) [128/178]
Caudal	90.5% (77.4%–97.3%) [38/42]	85.3% (78.9%–90.3%) [139/163]	88.1% (74.4%–96.0%) [37/42]	92.0% (86.7%–95.7%) [150/163]	92.9% (80.5%–98.5%) [39/42]	87.7% (81.7%–92.3%) [143/163]
Cervical interlaminar	82.8% (75.1%–88.9%) [106/128]	79.8% (74.6%–84.4%) [221/277]	82.8% (75.1%–88.9%) [106/128]	81.9% (76.9%–86.3%) [227/277]	98.4% (94.5%–99.8%) [126/128]	84.1% (79.3%–88.2%) [233/277]
Cervical transforaminal	92.3% (64.0%–99.8%) [12/13]	80.0% (61.4%–92.3%) [24/30]	84.6% (54.6%–98.1%) [11/13]	86.7% (69.3%–96.2%) [26/30]	92.3% (64.0%–99.8%) [12/13]	90.0% (73.5%–97.9%) [27/30]
Cervical facet	100.0% (2.5%–100.0%) [1/1]	75.0% (19.4%–99.4%) [3/4]	100.0% (2.5%–100.0%) [1/1]	100.0% (39.8%–100.0%) [4/4]	100.0% (2.5%–100.0%) [1/1]	100.0% (39.8%–100.0%) [4/4]
Others	100.0% (75.3%–100.0%) [13/13]	75.0% (57.8%–87.9%) [27/36]	92.3% (64.0%–99.8%) [12/13]	80.6% (64.0%–91.8%) [29/36]	100.0% (75.3%–100.0%) [13/13]	77.8% (60.8%–89.9%) [28/36]

Data are expressed as point estimates with 95% confidence intervals in parentheses and numerators/denominators in brackets. The cutoff values predicting SCID after spinal injection were established as follows: ≥ 3 for NRS absolute reduction, ≥ 50% for NRS relative reduction, and ≥50% for patient-reported relative reduction. NRS, numerical rating scale

## Discussion

We determined the cutoff values for clinically meaningful pain improvement after spinal injection using a large cohort of patients who received spinal injections. The thresholds for SCID (GPE 1–2) were established as an NRS absolute reduction of ≥3, an NRS relative reduction rate of ≥50%, and a PRR rate of ≥50%. Similarly, an NRS absolute reduction of ≥1, an NRS relative reduction rate of ≥10%, and a PRR rate of ≥20% were associated with MCID (GPE 1–3). Meanwhile, an NRS absolute reduction of ≥5, an NRS relative reduction rate of ≥90%, and a PRR rate of ≥90% corresponded to complete pain resolution (GPE 1). Among the three measures tested (i.e., NRS absolute reduction, NRS relative reduction, and PRR), PRR demonstrated the highest diagnostic performance in predicting clinically meaningful pain improvement, with an AUC of 0.959–0.998. The cutoff values for predicting SCID (GPE 1–2) demonstrated consistent sensitivity and specificity across various subgroups, including pain site with maximal NRS reduction and the type of spinal injection.

To the best of our knowledge, this study is the first to propose the criteria for clinically meaningful pain reduction after spinal injections and to evaluate their diagnostic performance. We evaluated not only low back pain and posterior neck pain but also pain radiating to the legs and arms, which should be carefully evaluated in spinal pathologies. Moreover, we proposed different cutoff levels by refining the definition of “clinically meaningful pain improvement.”

The mean NRS absolute reduction in the GPE 3 subgroup (slightly improved) was 2.4. A systematic review of the MCID for chronic pain reported a median NRS absolute reduction of 2.8 for back pain with radiculopathy and neck pain, which is similar to our findings [24]. This underscores that the mean NRS absolute reduction for low back pain and neck pain was similar among patients who received spinal injections. Furthermore, we presented the mean NRS relative reduction and mean PRR rates, categorized by GPE scores, to provide a basis for future researchers to establish benchmark values according to their preferences. In the GPE 2 subgroup (much improved), the NRS relative reduction rate was 65.4%, whereas the PRR rate was 67.7%.

Our cutoff values align with those used in previous randomized controlled studies [9]. Notably, the NRS absolute reduction of ≥3 and NRS relative reduction rate of ≥50%, which are cutoff values employed in previous studies, corresponded to our defined threshold for SCID. Although the rationale for establishing the 50% threshold in these randomized controlled studies was not explicitly discussed in previous literature, our findings support the clinical appropriateness of these criteria. In the context of spinal injections, researchers prefer to measure the rate of significant changes in pain following the procedure rather than focusing solely on minimal changes [9]. Hence, future studies should continue to adopt cutoff values that align with the SCID.

By contrast, the cutoff values we established for SCID were slightly higher. Conversely, the cutoff values for MCID were slightly lower than those suggested by a recent consensus statement [25] and for treatments other than spinal injection for low back pain or posterior neck pain. Notably, Ostelo et al. proposed that an NRS absolute reduction of ≥2 or an NRS relative reduction rate of ≥30% could indicate a treatment response for low back pain [25]. However, the definition of “improvement” varied across the studies cited by Ostelo et al.: one defined it as “much improved” and more effective based on a 5-point global change scale [26], while others either defined it as more than “slightly improved” and more effective [27] or used both criteria [7]. Additionally, the definitions can vary based on whether NRS reductions are considered absolute [26–28] or relative [7].

The PRR demonstrated the highest diagnostic performance among the tested measures for predicting clinically meaningful pain reduction. This finding is consistent with the reports of previous studies, which reported that the PRR is more accurate than the NRS [29]. Owing to the high intra-individual variability of the NRS, it is not uncommon to observe a higher NRS score after spinal injections, even when a patient reports a positive treatment response and vice versa. Although the NRS and visual analog scale are frequently used to assess pain intensity, the PRR or GPE would be a reliable option for evaluating changes in pain levels after spinal injection rather than simply interpreting NRS reduction. Nonetheless, this finding should be interpreted with caution because PRR and GPE represent retrospective patient-reported assessments of change after treatment. The conceptual similarity between PRR and GPE may have contributed to the higher diagnostic performance of PRR when GPE was used as the reference standard.

Our cutoff values demonstrated consistent sensitivity and specificity across various subgroups. These values could be utilized to evaluate the pain improvement after spinal injections for posterior neck pain, low back pain, and radiating pain. Moreover, no significant differences in diagnostic performance were observed across age, sex, or type of spinal injection, suggesting that our cutoff values can be widely applied to diverse populations and injection types. However, the sensitivity of NRS absolute reduction was lower in patients with a mild initial NRS score. This limitation is likely inherent in the NRS absolute reduction method, as interpreting an absolute reduction of ≥3 as a pain improvement in patients with an initial NRS score of ≤3 presents a contradiction.

Our study has some limitations. First, the generalizability of our results is limited owing to the single-center retrospective nature of this study, which was conducted only in the Korean population. Additionally, pain perception can vary according to ethnicity [30], and our findings may not be applicable to other ethnic groups. In addition, a substantial proportion of the initial cohort did not participate in the 2-week telephone interview, raising the possibility of non-response selection bias. Second, the applicability of our cutoff values to spinal injections performed for thoracic pathologies remains unclear due to the limited number of patients with thoracic pathologies or upper back pain included. Third, we did not assess the improvement in functional disability following spinal injections, although the therapeutic effect of spinal injection should be carefully evaluated not only in terms of pain reduction but also in terms of functional disability. Therefore, the cutoff values established in this study should be interpreted specifically as thresholds for clinically meaningful pain improvement and should not be used as sole indicators of overall treatment success after spinal injection. Fourth, our results might have differed had an alternative operational definition of GPE been used [24]. Fifth, the outcomes could have been influenced if the physician performing the procedure had conducted a face-to-face interview instead of relying on a questionnaire, although this remains debatable [31,32]. Finally, PRR was assessed retrospectively at the 2-week follow-up interview and may have been subject to recall bias.

In conclusion, we established the cutoff values for NRS absolute reduction, NRS relative reduction, and PRR to differentiate between SCID and MCID in terms of pain improvement after spinal injection. An NRS absolute reduction of ≥3, an NRS relative reduction rate of ≥50%, and a PRR rate of ≥50% were associated with SCID. Our findings may serve as a reference, aiding researchers in developing a more standardized framework for evaluating pain improvement after spinal injection.

## Supporting Information

S1 Appendix Patient Questionnaire. (DOCX)

## References

[1] Manchikanti L, Pampati V, Falco FJE, Hirsch JA. Assessment of the growth of epidural injections in the medicare population from 2000 to 2011. Pain Physician. 2013;16(4):E349-64. doi: 10.36076/ppj.2013/16/e349 23877459

[2] North RB, Campbell JN, James CS, Conover-Walker MK, Wang H, Piantadosi S, et al. Failed back surgery syndrome: 5-year follow-up in 102 patients undergoing repeated operation. Neurosurgery. 1991;28(5):685–90; discussion 690-1. doi: 10.1227/00006123-199105000-00008 1831546

[3] North RB, Ewend MG, Lawton MT, Kidd DH, Piantadosi S. Failed back surgery syndrome: 5-year follow-up after spinal cord stimulator implantation. Neurosurgery. 1991;28(5):692–9. doi: 10.1227/00006123-199105000-00009 1831547

[4] Seres JL. The fallacy of using 50% pain relief as the standard for satisfactory pain treatment outcome. Pain Forum. 1999;8(4):183–8. doi: 10.1016/s1082-3174(99)70005-x

[5] Rowbotham MC. What is a “clinically meaningful” reduction in pain?. Pain. 2001;94(2):131–2. doi: 10.1016/S0304-3959(01)00371-2 11690725

[6] Farrar JT, Portenoy RK, Berlin JA, Kinman JL, Strom BL. Defining the clinically important difference in pain outcome measures. Pain. 2000;88(3):287–94. doi: 10.1016/S0304-3959(00)00339-0 11068116

[7] Farrar JT, Young JP Jr, LaMoreaux L, Werth JL, Poole MR. Clinical importance of changes in chronic pain intensity measured on an 11-point numerical pain rating scale. Pain. 2001;94(2):149–58. doi: 10.1016/S0304-3959(01)00349-9 11690728

[8] Cepeda MS, Africano JM, Polo R, Alcala R, Carr DB. What decline in pain intensity is meaningful to patients with acute pain?. Pain. 2003;105(1–2):151–7. doi: 10.1016/s0304-3959(03)00176-3 14499431

[9] Manchikanti L, Knezevic NN, Boswell MV, Kaye AD, Hirsch JA. Epidural Injections for Lumbar Radiculopathy and Spinal Stenosis: A Comparative Systematic Review and Meta-Analysis. Pain Physician. 2016;19(3):E365-410. doi: 10.36076/ppj/2016.19.e365 27008296

[10] Zhao W, Wang Y, Wu J, Gao X, Wei Q, Lai X, et al. Long-Term Outcomes of Epidurals with Lidocaine With or Without Steroids for Lumbar Disc Herniation and Spinal Stenosis: A Meta-Analysis. Pain Physician. 2020;23(4):365–74. doi: 10.36076/ppj.2020/23/365 32709171

[11] Manchikanti L, Nampiaparampil DE, Manchikanti KN, Falco FJE, Singh V, Benyamin RM, et al. Comparison of the efficacy of saline, local anesthetics, and steroids in epidural and facet joint injections for the management of spinal pain: A systematic review of randomized controlled trials. Surg Neurol Int. 2015;6(Suppl 4):S194-235. doi: 10.4103/2152-7806.156598 26005584 PMC4431057

[12] Manchikanti L, Knezevic NN, Parr A, Kaye AD, Sanapati M, Hirsch JA. Does Epidural Bupivacaine with or Without Steroids Provide Long-Term Relief? A Systematic Review and Meta-analysis. Curr Pain Headache Rep. 2020;24(6):26. doi: 10.1007/s11916-020-00859-7 32335757

[13] Knezevic NN, Manchikanti L, Urits I, Orhurhu V, Vangala BP, Vanaparthy R, et al. Lack of Superiority of Epidural Injections with Lidocaine with Steroids Compared to Without Steroids in Spinal Pain: A Systematic Review and Meta-Analysis. Pain Physician. 2020;23(4S):S239–70. doi: 10.36076/ppj.2020/23/s239 32942786

[14] Manchikanti L, Atluri S, Boswell MV, Calodney AK, Diwan S, Gupta S, et al. Methodology for Evidence Synthesis and Development of Comprehensive Evidence-Based Guidelines for Interventional Techniques in Chronic Spinal Pain. Pain Physician. 2021;24(S1):S1–26. doi: 10.36076/ppj.2021.24.s1-s26 33492917

[15] McGlothlin AE, Lewis RJ. Minimal clinically important difference: defining what really matters to patients. JAMA. 2014;312(13):1342–3. doi: 10.1001/jama.2014.13128 25268441

[16] Cook CE. Clinimetrics Corner: The Minimal Clinically Important Change Score (MCID): A Necessary Pretense. J Man Manip Ther. 2008;16(4):E82-3. doi: 10.1179/jmt.2008.16.4.82E 19771185 PMC2716157

[17] Patel IJ, Davidson JC, Nikolic B, Salazar GM, Schwartzberg MS, Walker TG, et al. Addendum of newer anticoagulants to the SIR consensus guideline. J Vasc Interv Radiol. 2013;24(5):641–5. doi: 10.1016/j.jvir.2012.12.007 23622037

[18] Manchikanti L, Kaye AD, Soin A, Albers SL, Beall D, Latchaw R, et al. Comprehensive Evidence-Based Guidelines for Facet Joint Interventions in the Management of Chronic Spinal Pain: American Society of Interventional Pain Physicians (ASIPP) Guidelines Facet Joint Interventions 2020 Guidelines. Pain Physician. 2020;23(3S):S1–127. doi: 10.36076/ppj.2020/23/s1 32503359

[19] Bogduk N, MacVicar J, Borowczyk J. The pain of vertebral compression fractures can arise in the posterior elements. Pain Med. 2010;11(11):1666–73. doi: 10.1111/j.1526-4637.2010.00963.x 21044256

[20] Wilson DJ, Owen S, Corkill RA. Facet joint injections as a means of reducing the need for vertebroplasty in insufficiency fractures of the spine. Eur Radiol. 2011;21(8):1772–8. doi: 10.1007/s00330-011-2115-5 21487701

[21] Shim E, Lee JW, Lee E, Ahn JM, Kang Y, Kang HS. Fluoroscopically Guided Epidural Injections of the Cervical and Lumbar Spine. Radiographics. 2017;37(2):537–61. doi: 10.1148/rg.2017160043 27935769

[22] Kim Y, Lee E, Lee JW, Kang Y, Ahn JM, Kang HS. Clinical and imaging characteristics of patients with extreme low back pain or sciatica referred for spinal injection. Neuroradiology. 2019;61(8):881–9. doi: 10.1007/s00234-019-02222-z 31101947

[23] DeLong ER, DeLong DM, Clarke-Pearson DL. Comparing the areas under two or more correlated receiver operating characteristic curves: a nonparametric approach. Biometrics. 1988;44(3):837–45. doi: 10.2307/2531595 3203132

[24] Olsen MF, Bjerre E, Hansen MD, Tendal B, Hilden J, Hróbjartsson A. Minimum clinically important differences in chronic pain vary considerably by baseline pain and methodological factors: systematic review of empirical studies. J Clin Epidemiol. 2018;101:87-106.e2. doi: 10.1016/j.jclinepi.2018.05.007 29793007

[25] Ostelo RWJG, Deyo RA, Stratford P, Waddell G, Croft P, Von Korff M, et al. Interpreting change scores for pain and functional status in low back pain: towards international consensus regarding minimal important change. Spine (Phila Pa 1976). 2008;33(1):90–4. doi: 10.1097/BRS.0b013e31815e3a10 18165753

[26] van der Roer N, Ostelo RWJG, Bekkering GE, van Tulder MW, de Vet HCW. Minimal clinically important change for pain intensity, functional status, and general health status in patients with nonspecific low back pain. Spine (Phila Pa 1976). 2006;31(5):578–82. doi: 10.1097/01.brs.0000201293.57439.47 16508555

[27] Grotle M, Brox JI, Vøllestad NK. Concurrent comparison of responsiveness in pain and functional status measurements used for patients with low back pain. Spine (Phila Pa 1976). 2004;29(21):E492-501. doi: 10.1097/01.brs.0000143664.02702.0b 15507789

[28] Childs JD, Piva SR, Fritz JM. Responsiveness of the numeric pain rating scale in patients with low back pain. Spine (Phila Pa 1976). 2005;30(11):1331–4. doi: 10.1097/01.brs.0000164099.92112.29 15928561

[29] Seo J, Lee JW, Kang Y, Lee E, Ahn JM, Kim DH, et al. Evaluation of the responsiveness of outcome measures after spine injection: A retrospective study. PLoS One. 2019;14(2):e0211763. doi: 10.1371/journal.pone.0211763 30811428 PMC6392275

[30] Campbell CM, Edwards RR. Ethnic differences in pain and pain management. Pain Manag. 2012;2(3):219–30. doi: 10.2217/pmt.12.7 23687518 PMC3654683

[31] Holmström E, Moritz U. Low back pain--correspondence between questionnaire, interview and clinical examination. Scand J Rehabil Med. 1991;23(3):119–25. doi: 10.2340/165019779123119125 1835797

[32] Horton L, Rhodes J, Menon DK, Maas AIR, Wilson L, Collaborative European NeuroTrauma Effectiveness Research in TBI (CENTER-TBI) Participants and Investigators. Questionnaires vs Interviews for the Assessment of Global Functional Outcomes After Traumatic Brain Injury. JAMA Netw Open. 2021;4(11):e2134121. doi: 10.1001/jamanetworkopen.2021.34121 34762111 PMC8586906

