# Supplementary material for: Defining clinically meaningful pain reduction after spinal injections using an 11-point numerical rating scale and patient-reported relative reduction of pain
Source: PLoS One. 2026 Oct 26;21(10):e0361475. doi: 10.1371/journal.pone.0361475 (PMC13704859; doi:10.1371/journal.pone.0361475)
Supplement: Supplementary file 1 [file pone.0361475.s001.docx]

**S1 Appendix. Patient Questionnaire**

This questionnaire is intended to evaluate your condition for an injection procedure. Please fill in the information below.

1) **Date :**
2) **Duration of pain onset :**
3) **Pain Score (NRS, 0–10)**

- **Lower back pain score (0–10) :**
- **Leg pain score (0–10) :**
- **Posterior neck pain (0–10) :**
- **Arm pain (0–10) :**
- **Upper back pain (0–10) :**
